# Carbolic Acid as a Local Anæsthetic

**Published:** 1883-07

**Authors:** 


### CARBOLIC ACID AS A LOCAL ANÆSTHETIC.

Dr. A. M. Pelton says, in the Southern Practitioner: By the following method abscesses, felons, boils, etc., can be opened with little or no pain. Sharpen to a point a stick about six inches in length. Dip the point into liquified carbolic acid, and apply to the point chosen for the opening. After a moment's delay, cut the skin with a knife; then take a little of the acid on the point of the stick and apply in the incision with a gentle rotary motion. By frequent applications of the acid, and a gentle rotary motion of the stick persistently applied, an opening can be made to the required depth. The carbolic acid produces first anæsthesia, then death of the parts to which it is applied in the foregoing manner.

